# Unraveling Audiovisual Perception Across Space and Time: A Neuroinspired Computational Architecture

**DOI:** 10.1111/ejn.70217

**Published:** 2025-08-05

**Authors:** Cristiano Cuppini, Eleonore F. Di Rosa, Laura Astolfi, Melissa Monti

**Affiliations:** ^1^ Department of Electrical Electronic and Information Engineering “Guglielmo Marconi” (DEI), University of Bologna Bologna Italy; ^2^ Department of Translational Neuroscience Wake Forest University School of Medicine Winston‐Salem North Carolina USA; ^3^ Department of Computer, Control and Management Engineering University of Rome La Sapienza Rome Italy

**Keywords:** audiovisual processing, computational modelling, multisensory integration, spatial ventriloquism, switch cost

## Abstract

Accurate perception of audiovisual stimuli depends crucially on the spatial and temporal properties of each sensory component, with multisensory enhancement only occurring if those components are presented in spatiotemporal congruency. Although spatial localization and temporal detection of audiovisual signals have each been extensively studied, the neural mechanisms underlying their joint influence, particularly in spatiotemporally misaligned contexts, remain poorly understood. Moreover, empirical dissection of their respective contributions to behavioral outcomes proves challenging when spatial and temporal disparities are introduced concurrently. Here, we sought to elucidate the mutual interaction of temporal and spatial offsets on the neural encoding of audiovisual stimuli. To this end, we developed a biologically inspired neurocomputational model that reproduces behavioral evidence of perceptual phenomena observed in audiovisual tasks, i.e., the modality switch effect (temporal realm) and the ventriloquist effect (spatial realm). Tested against the race model, our network successfully simulates multisensory enhancement in reaction times due to the concurrent presentation of cross‐modal stimuli. Further investigation on the mechanisms implemented in the network upheld the centrality of cross‐sensory inhibition in explaining modality switch effects and of cross‐modal and lateral intra‐area connections in regulating the evolution of these effects in space. Finally, the model predicts an amelioration in temporal detection of different modality stimuli with increasing between‐stimuli eccentricity and indicates a plausible reduction in auditory localization bias for increasing interstimulus interval between spatially disparate cues. Our findings provide novel insights into the neural computations underlying audiovisual perception and offer a comprehensive predictive framework to guide future experimental investigations of multisensory integration.

## Graphical abstract

Fitted on auditory localization and reaction time task data, our neurocomputational model aims to elucidate the mechanisms underlying multisensory perception in the entire spatiotemporal domain, predicting how spatial and temporal factors interact to modulate sensory perception. A is for Auditory, V for Visual, AV for Audiovisual, Sw for a trial comprising two sequential stimuli of different sensory modalities, Rp for the same sensory modality (e.g., in SwA, V is followed by A).

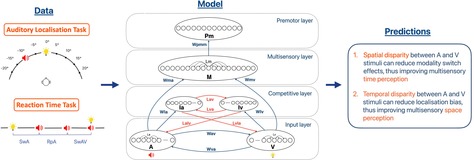

**Abbreviations**
A: auditory
AV: audiovisual
CDF: cumulative distribution function
Ia: acoustic interneurons
ISI: interstimulus‐interval
Iv: visual interneurons
M: multisensory
PM: premotor
Rp: repeat
RSE: redundant signals effect
RT: reaction time
Sw: switch
V: visual

## 1 Introduction

In everyday life, we encounter a multiplicity of sensory inputs, such as lights and sounds that come at us in an endless stream. Our brain has therefore evolved to process each sensory signal and combine their content to react appropriately in every situation, according to the synergistic ensemble of mechanisms also known as multisensory integration (Stein and Stanford 2008). This capability develops thanks to experience during early postnatal life (Stein et al. 2014) and critically depends on a variety of factors, including the spatial and temporal relationships among cross‐modal stimuli (Frens et al. 1995; Stein 2012; Wallace and Stein 2007; Xu et al. 2012, 2014).

Several neurophysiological studies showed that the response of a multisensory neuron to a pair of spatiotemporally congruent cross‐modal stimuli is significantly enhanced as compared to those evoked by either of the modality‐specific stimuli (Stein and Meredith 1993). However, introducing a spatial discrepancy between the sensory components of a cross‐modal stimulus may prevent the multisensory neuron from response enhancement, or even lead to response depression (Meredith and Stein 1996). A similar result is observed when the sensory components of the stimulus are delivered congruent in space but temporally misaligned, for interstimulus intervals (ISIs) exceeding 200–400 ms (Diederich and Colonius 2004a; Meredith et al. 1987). The physiological changes that derive from response enhancement or depression produce perceptual and behavioral alterations. Spatiotemporal congruency between cues has thus been found responsible for higher accuracy in detection and localization, faster reaction times (RTs), and ameliorated decision‐making (Bolognini et al. 2007; Diederich and Colonius 2004a; Senkowski et al. 2007; von Saldern and Noppeney 2013).

Considerable research effort has been devoted to detailing separately the spatial or the temporal implications of the most diverse integrative phenomena, under different experimental conditions and adopting various computational strategies (for studies on the temporal dimension, see Colonius and Diederich 2020; Mégevand et al. 2013; Musacchia and Schroeder 2009; Navarra et al. 2005; Rowland et al. 2007; Rowland and Stein 2007; Stevenson and Wallace 2013; Zhou et al. 2020; for studies on the spatial dimension, see Alais and Burr 2004; Bertelson and Radeau 1981; Jones and Noppeney 2024; Meredith and Stein 1996; Odegaard et al. 2015; Thurlow and Jack 1973; Wozny et al. 2010; see Recanzone 2009 for a review). Significantly few works, however, have addressed multisensory perception in space *and* time‐varying conditions, and even fewer have tried to provide a plausible description of the neural correlates that subtend the processing and combination of such cross‐modal relationships. Examples of experimental paradigms can be found in literature in which cross‐modal stimuli, especially auditory and visual, are presented at a variety of spatiotemporal disparities (Lewald et al. 2001; Lewald and Guski 2003; Wallace et al. 2004). However, the vast majority of them eventually analyses the effect of cross‐modal stimulation on the representation of sensory space and on target localization, without providing a similar description of how temporal detection performance might be affected by the same stimuli configurations. Neurocomputational approaches have always played a central role in the understanding of brain integrative phenomena. In particular, adopting biologically plausible models helps in suggesting the hypothetical synaptic architecture that subtends the multisensory processing of cross‐modal stimuli delivered in different spatiotemporal configurations (Ursino et al. 2014). For instance, Cuppini et al. (2020) proposed a potential description for a competitive architecture among auditory and visual processing systems to explain the so‐called modality switch effect: Shaw et al. (2020) and Crosse et al. (2022) showed that reacting to sensory stimuli takes longer when modality switches, rather than when it repeats. Since this work mainly aimed at characterizing the variation of the switch cost along the temporal coordinate, and at predicting which synaptic connections mediate cross‐sensory inhibition at the neural level, the structure of the model did not allow for also investigating how the switch cost may vary along the spatial coordinate, nor the neural underpinnings that might be involved in this case.

The aim of this research is therefore to propose a novel biologically inspired neurocomputational model that builds upon time‐oriented and space‐oriented previous modelling works (Cuppini et al. 2017; Cuppini et al. 2020), in order to (1) formulate predictions on the spatial characterization of modality switch effects, (2) observe the potential influence of cross‐sensory inhibition on the localization of sensory targets, and finally (3) create a comprehensive theoretical framework for the study of multisensory behavior in the spatiotemporal domain.

## 2 Materials and Methods

The model was realized by merging and improving two previous models (Cuppini et al. 2020 and Cuppini et al. 2017), and it was implemented in MATLAB (The MathWorks Inc. 2024). The former aimed at simulating data from a simple RT task, with auditory and visual stimuli presented at different ISIs but no spatial disparity (Crosse et al. 2022). On the other hand, the latter was designed to identify the likely neural mechanisms involved in the spatial detection of auditory stimuli presented along with synchronous visual stimuli at different locations in space. To this aim, researchers simulated behavioral experiments of sensory detection tasks and compared the results with data from the literature (Bertelson and Radeau 1981; Odegaard et al. 2015; Rohe and Noppeney 2015b). In the following, we will provide a qualitative description of the model, while the accurate mathematical description is presented in the Supporting Information.

### 2.1 Qualitative Description of the Model

Each area is composed of 180 neural units, assumed at 1° of distance from one another, and each is responsive to a specific portion of the external space. Computationally, each unit is described by a first‐order dynamics, simulating the integrative properties of the cellular membrane. The I/O relationship of each unit is sigmoidal, thus allowing (1) the simulation of neural activation with a lower threshold and upper saturation, and (2) the possibility to easily elicit a suprathreshold activity in silent neurons that activate as a result of noise/cross‐modal influences. The saturation value has been set at 1, so all outputs are normalized to the maximum.

**FIGURE 1:**
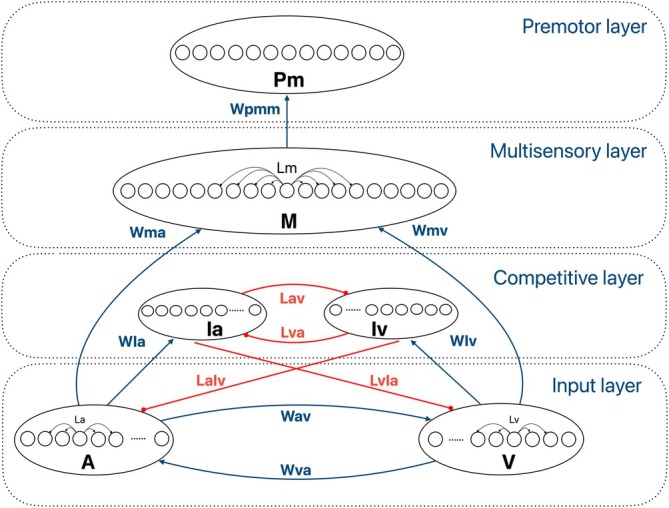
Model structure: A and V represent the auditory and visual areas, responsible for the first sensory processing and the generation of localization biases. They exchange direct cross‐modal excitatory synapses, Wav and Wva. The multisensory area M receives feedforward excitatory projections from unisensory areas, Wma and Wmv, integrates the result of unisensory processing, and excites the premotor region via Wpmm. The premotor region pm generates the simulated RTs to external stimuli. The unisensory interneural areas Iv and Ia are excited by the input layer via the connections WIa and WIv, and they implement a competitive mechanism between modalities via winner‐takes‐all dynamics, mediated by the connections Lav and Lva among Ia nad Iv and projected back to the unisensory areas via the inhibitory connections LaIv and LvIa. Lv, La, and Lm are the intra‐area lateral connections within the V, A, and M areas, respectively. Blue lines represent excitatory connections; red lines represent inhibitory connections.

The model is built upon four layers:

1 The **input layer**: composed of two regions, topographically organized, that represent unisensory visual and auditory input areas (V and A in Figure 1), respectively. While a topographic organization of visual space is known to be already present in the primary visual cortex, several studies in humans and primates showed that auditory neurons sensitive to specific sound locations are present in the planum temporale and superior temporal gyrus, so that spatial representations of sounds eventually emerge along the dorsal processing stream (Kuśmierek and Rauschecker 2014; Miller and Recanzone 2009; Zatorre et al. 2002; Zimmer and Macaluso 2005; Zündorf et al. 2013). A detailed representation of the primary auditory cortex is not necessary for the aims of the present model, but these findings allow us to interpret the activity of each neural unit in the auditory input area of this model as the functional result of auditory processing along the dorsal stream.

Each neural unit in areas A and V receives four inputs:

- An external modality‐specific input, representing an incoming stimulus from the outside world, simulated by a Gaussian function and filtered by the receptive fields of the neural units. The central point of the Gaussian coincides with the application point of the stimulus in the external world, while the combined effect of the width of unisensory neurons' RFs and the reliability of the external inputs is represented in the standard deviation (*σ*_a_ and *σ*_v_). As shown in Ursino et al. 2017, both terms influence the perceived position of neural inputs. The onset and the duration were chosen to reproduce the experimental setup of Crosse et al. (2022). To address experimental variability, the effectiveness of external inputs (I_0_^v^ and I_0_^a^) was randomly chosen from a uniform distribution (see Table 1).
- A lateral input from elements in the same regionthat is excitatory for proximal units and inhibitory for distal units is mediated by intra‐area synapses (L^v^ and L^a^), having the shape of a Mexican hat. This type of connectivity pattern has been extensively used to account for orientation and direction selectivity of visual neurons, but recent studies have advanced the hypothesis that similar centre‐surround dynamics might also be employed by the auditory system to encode spectral features that might contribute to sound localization (Gilday et al. 2023; Lakunina et al. 2020).
- A cross‐modal input from the corresponding units in the other input region. Neural units that cover adjacent portions of the external space in the two regions exchange excitatory cross‐modal synapses (Wav and Wva in Figure 1), characterized by Gaussian distribution and fast dynamics. The effectiveness of these connections is strong enough to induce significant increases in the activity of the other sensory area in case of multisensory stimulation, but not in unisensory conditions. This is in line with numerous findings from animal studies (see Ghazanfar and Schroeder 2006; Opoku‐Baah et al. 2021; Teichert and Bolz 2018 for reviews).
- An inhibitory input from the interneural units of the other sensory modality is mediated by Gaussian‐shaped inhibitory feedback synapses with a slow dynamic (LaIv and LvIa in Figure 1). When different modality‐specific stimuli are presented sequentially, this input creates a cross‐sensory competition that eventually translates into the switch cost observed at the behavioral level. A growing body of studies suggests that both cortical (Iurilli et al. 2012; Lemercier et al. 2024) and subcortical (Chou et al. 2020) pathways may be involved in cross‐sensory modulation between audition and vision and that temporal discrepancy between cross‐modal stimuli is likely to result in suppressive interactions between sensory systems (Meijer et al. 2017).

**TABLE 1:** Parameters values for the single neural unit, for the sensory inputs, and for the inhibitory dynamics. A brief description of the single neural units is provided in Subsection 2.1. Incoming signals to each area, i.e., the inputs, are described in Subsection 2.3 Parameter Assignment. The term “Inhibition” refers generally to connections carrying negative‐signed contributions to the activity of a target area.

Neural unit			
dim = 100	θ = 25	s = 0.3	τ = 3 ms

Inputs			
G^r^_i_ = 75	i = e, m, pm	τ^r^_i_ = 15 ms	i = I_ex, I_in
τ^a^_e_ = 15 ms	τ^v^_e_ = 25 ms	τ_cross_ = 3 ms	
τ^r^_i_ = 3 ms	i = m, pm		
I^a^_0_ = [1.35–1.97]	I^v^_0_ = [1.8–2.6]	*σ*_a_ = 32	*σ*_v_ = 4
Δt = 16 ms	Δt^m^ = 50 ms	AV ratio = 3/5	

Inhibitory dynamics			
G^r^_l_ = 750	r = a, v	τ^r^_l_ = 180 ms	r = a, v

The perceived position of the external stimuli is inferred in the input regions; in particular, it is computed as the barycentre of the activities elicited in each area. Due to the presence of cross‐modal projections, the inferred spatial localization of the auditory or visual inputs is affected by the concurrent presentation of the stimulus in the other sensory modality, even if the two unisensory events are processed separately. Excitatory Gaussian‐shaped synapses (WIa and WIv) project from the input areas towards modality‐specific interneural areas (Ia and Iv) that are part of the competitive layer described below, and long‐range excitatory connections (Wma and Wmv in Figure 1) project towards a multisensory area (M region in Figure 1).

2 The **competitive layer**: It is formed by two areas of interneural units, one for each modality. Elements in these areas are excited by elements from the same‐modality input area, as well as an inhibitory input from the other interneural area, according to a winner‐takes‐all competitive mechanism. With this structure, the “winner” sensory modality inhibits the other modality through the feedback inhibitory synapses LaIv and LvIa that communicate with the unisensory regions. This competitive layer may find physiological implementation in higher order regions, as suggested by Huang et al. 2015 and Hairston et al. 2008 in an auditory temporal order judgment task. Nonetheless, direct projections between core visual and auditory areas and associative cortices (Clavagnier et al. 2004), or functional sensory modulation (Bizley and King 2009; Yu et al. 2013) may also be responsible for this kind of competition.
3 The **multisensory** area: Units in this area receive Gaussian‐shaped excitatory feedforward synapses (Wmv and Wma) from elements of the unisensory areas that are sensitive to the same portion of the space, and a lateral input, generated by other units in the region, via Mexican hat intra‐area synapses (Lm). Regarding the biological plausibility of this region, several studies have demonstrated that associative areas such as the inferior parietal lobule and the superior temporal sulcus receive converging inputs from multiple unisensory regions, including visual cortices, the planum temporale, and the superior temporal gyrus. These areas are critically involved in multisensory integration and sensorimotor processing (Beauchamp 2005; Calvert et al. 2001; Ghazanfar and Schroeder 2006).
4 The **premotor** area, in which each neural unit is connected to the corresponding unit in the multisensory area, is used to simulate the behavioral responses of subjects to external stimuli, and its elicited activity is compared with a fixed threshold (30% of the maximum neurons' activity) to evaluate RTs to the external stimuli.

### 2.2 Parameter Assignment

The values of model parameters (see Tables 1 and 2) were chosen based on data from literature and from Cuppini et al. (2020), considering that the only addition to the model architecture is the premotor area, and that differently from Cuppini et al. (2020), each area in the present model consists of *N* = 180 elements to account for the spatial dimension. Consequently, the synaptic connections are now modelled with a Gaussian distribution, as in Cuppini et al. (2017).

**TABLE 2:** Parameters values of the effectiveness (subscript 0) and standard deviation (subscript SD) of the connections included in the model. Label definitions for each connection are provided in the caption of Figure 1, and further detailed in the Supporting Information.

Connections				
(1) Feedforward to multisensory				
Wma_0_ = 0.12	Wmv_0_ = Wma_0_/AV ratio		Wma_SD_ = Wmv_SD_ = 5	
(2) Cross‐modal				
Wva_0_ = 0.12	Wav_0_ = Wva_0_/AV ratio		Wav_SD_ = 7	Wva_SD_ = 5
(3) Intra‐area Mexican hat lateral inhibition				
(a) Multisensory				
LLm_in_0_ = 0.3	LLm_ex_0_ = 0.4		LLm_in_SD_ = 8	LLm _ex_SD_ = 4
(b) Auditory				
LLa_in_0_ = 4	LLa_ex_0_ = 5		LLa_in_SD_ = 120	LLa_ex_SD_ = 3
(c) Visual				
LLv_in_0_ = 4	LLv_ex_0_ = 5		LLv_in_SD_ = 120	LLv_ex_SD_ = 3
(4) Feedforward excitatory to Interneurons				
WIa_0_ = 0.2	WIv_0_ = WIa_0_/AV ratio		WIa_SD_ = WIv_SD_ = 5	
(5) Feedback inhibition from interneurons to primary areas				
LaIv_0_ = 0.0057	LvIa_0_ = 0.016		LaIv_SD_ = LvIa_SD_ = 15	
(6) WTA between interneurons—1 to 1 connectivity				
Lva0 = Lav0 = 10				
(7) Feedforward to premotor area—1 to 1 connectivity				
Wpmm_0_ = 1				

#### 2.2.1 Individual Neural Units

The central abscissa, θ, was chosen to produce negligible activation when the input is null. The slope of the sigmoidal relationship, s, is responsible for a smooth transition from null activity to saturation in response to external stimuli. The time constant agrees with values usually found in deterministic mean‐field equations (Ben‐Yishai et al. 1995; Treves 1993).

#### 2.2.2 External Input $e_{j}^{r}\left(t\right)$

Physiological studies proved that auditory cortical neurons exhibit shorter latencies (e.g., Recanzone et al. 2000) than neurons in the visual cortex (Maunsell and Gibson 1992). Therefore, the time constant was chosen slower for the visual stimulus than for the auditory stimulus ($\tau^{a}$ <$\;\tau^{v}$). In particular, $\tau^{a}$ reproduces an activation of the auditory area occurring after 25–30 ms from the external stimulus onset. Since time constants represent the needed time for the activity in the input regions to reach 90% of their steady‐state value in response to a step input, we chose $\tau^{a}$ = 15 ms. On the other hand, $\tau^{v}$ was set at 25 ms, to simulate the effect of a visual stimulus producing a detectable response 45–50 ms after its onset. The visual system is also known to present better spatial resolution than the auditory one (see Recanzone 2009; Recanzone et al. 2000). Therefore, $\sigma^{v}$ was set to mimic sharper perception in the visual stream (which means the ability to detect the correct spatial position of an external cue with a few degree of uncertainty), while $\sigma^{a}$ was set greater than $\sigma^{v}$, according to observations of a broader spatial tuning of auditory neurons (Middlebrooks et al. 2002) and to previous computational studies (Cuppini et al. 2014; Magosso et al. 2012). The strength of the external visual and auditory stimuli (parameters Iv and Ia) was chosen to elicit a response, in the input regions in the upper portion of the sigmoidal static characteristic, that is close to saturation.

#### 2.2.3 Lateral Intra‐Area Component $l_{j}^{r}\left(t\right)$

$L_{\mathrm{ex}0}^{r}$, $L_{\text{in}0}^{r}$, $\sigma_{\mathrm{ex}}^{r}$, $\sigma_{\text{in}}^{r}$ were assigned to grant balance between excitation and inhibition and thus avoid instability, i.e., an uncontrolled excitation, which propagated to the overall area, and to allow a strong competition between two inputs of the same sensory modality. Conversely, the width of inhibitory synapses within the multisensory area is smaller, to enable the discrimination of unrelated events.

#### 2.2.4 Inhibitory Component ${\mathrm{Li}}_{j}^{r}\left(t\right)$

To simulate a strongest cross‐sensory inhibitory effect at ISIs close to 1000 ms (Crosse et al. 2022), we chose a slow dynamics for inhibitory mechanisms, implemented by time constants for the feedback projections equal to 180 ms ($\tau_{\text{in}}^{a}$ =$\;\tau_{\text{in}}^{v}$).

#### 2.2.5 Cross‐Modal Component $c_{j}^{r}\left(t\right)$

The dynamics of the cross‐modal term are symmetrical across sensory modalities. Their time constant (𝜏 = 3 ms) and the delay in cross‐modal synapses, ∆t = 16 ms, were selected so that the cross‐modal component produces an effect on “the other region” after 30–40 ms. These parameters allow to produce a rapid transient excitatory effect between input areas, as mounting evidence in the literature suggests happening as early as in the primary cortices (see Foxe and Schroeder 2005; Musacchia and Schroeder 2009; Recanzone 2009; Stein and Stanford 2008 for reviews).

#### 2.2.6 Interneural Excitatory Component $I_{\mathrm{ex}}^{r}\left(t\right)$

WI was chosen so that even a small activity in the input layer can activate the cross‐sensory inhibitory mechanism, by stimulating the corresponding interneural unit. The time constant $\tau_{\mathrm{Iex}}^{r}$ = 15 ms is the same as in previous models (Cuppini et al. 2014, 2020).

#### 2.2.7 Interneural Reciprocal Inhibitory Input $I_{\text{in}}^{r}\left(t\right)$

The effectiveness of the reciprocal synapses was selected so that the winner interneural unit is able to turn off almost completely the competing element. The time constant remained the same as previous models (Cuppini et al. 2014, 2020).

#### 2.2.8 Excitatory Input to the Multisensory Area ${\mathrm{ex}}^{m}\left(t\right)$

Wm was chosen so that even a small input activity, such as 30% of its saturation value, elicits a response in the output area a little below saturation. The delay ${\Delta t}^{m}$ (=50 ms), along with the activation delay ${\Delta t}^{\mathrm{pm}}\;\left(={\Delta t}^{m}\right)$ of the premotor area, was assigned in accordance with the threshold assumed by Crosse et al. 2022 to detect fast outliers (quicker RTs, less than 100 ms, are considered anticipatory responses). Together, this dynamic was included to simulate the neural process subtending the production of a motor response in the premotor area.

$G_{i}^{r}$ values (see Table 1) have been tuned so that the elicited activity in the postsynaptic units stays in the linear portion of the sigmoidal relationship.

The implementation of the connections along the two sensory pathways, A and V, is asymmetrical in the network: The effectiveness of the connections realizing the visual pathway (e.g., Wmv0, Wav0, and WIv0) is higher than the effectiveness of corresponding auditory connections (e.g., Wma0, Wva0, and WIa0). Motivations supporting our choice to model the visual stream as spatially sharper than the auditory one include (1) the well‐documented higher resolution and reliability of spatial information conveyed by the visual system, which, differently from auditory cues, is not spatially limited by the physical properties of sounds and the structure of the ears and head (Blauert 1996; King et al. 2001); (2) the higher computational demand to create a map of auditory space, which depends on precise integration, in time and space, of information from different frequency bands.

### 2.3 Assessment of the Network Performance

First, we assessed the multisensory performance of the model with two different sets of simulations, space‐oriented and time‐oriented, aimed respectively at reproducing results from Cuppini et al. (2017), for audiovisual integration in the spatial domain, and from Cuppini et al. (2020) for the multisensory benefits found in the temporal detection of modality switching stimuli.

#### 2.3.1 Time Domain

We presented to the model sequences of unisensory (auditory‐alone, A and visual‐alone, V) and multisensory stimuli (audiovisual inputs, AV) in a randomized order, at random ISIs between 1000 and 3000 ms, and aligned with central fixation (i.e., no spatial disparity), as in Cuppini et al. (2020). Simulated RTs were computed as the time interval between the instant of input presentation and the instant when the evoked activity in the output area reaches the perceptual threshold. Results were analyzed based on each input modality and the sensory modality of the previous input, discriminating between “Repeat” trials (the following stimulus is of the same sensory modality, Rp) and “Switch” trials (the following stimulus is of a different sensory modality, Sw). In all, RTs were simulated for the seven stimuli configurations presented in Crosse et al. (2022), that is, RpA, SwA, RpV, SwV, RpAV, SwA → AV, SwV → AV. The Audiovisual to Auditory and Audiovisual to Visual were excluded as they could be classified neither as Sw nor Rp. The effectiveness of each input was randomly chosen from a uniform distribution in order to replicate the within‐subject variability of sensory stimuli in a real environment. For every stimulus configuration, we computed the mean RTs over 100 simulations and performed a 3‐way ANOVA with Bonferroni correction for multiple comparisons with factors source (empirical data from NT adults from Crosse et al. (2022), simulated data from Cuppini et al. (2020), simulated data from the present model), condition (Rp and Sw) and sensory modality (A, V, and AV).

#### 2.3.2 Space Domain

We presented simultaneous AV stimuli to the network with different spatial disparity between the A and V components. The V stimulus was fixed at 0° (central fixation), while the position of the A stimulus was shifted, starting from 0° (i.e., A and V spatially coincident) and moving A to the left (and to the right) with a 5° eccentricity step, until a maximum eccentricity of 20° was reached (i.e., A placed at 0°, ±5°, ±10°, ±15°, and ±20°). For each stimulus configuration, the auditory perception bias was computed as the spatial disparity between the actual position of the external A stimulus and the position evaluated by the model (i.e., the barycenter of the evoked activity in the auditory area), divided by the distance between the actual A and the actual V stimulus positions. Because visual localization is only barely affected by sounds, the visual perception bias has not been reported. For every stimulus configuration, the mean Auditory Perception Bias was computed over 100 simulations, and results were then compared with those from Cuppini et al. (2017) and with empirical data (Bertelson and Radeau 1981; Rohe and Noppeney 2015b; Wallace et al. 2004).

#### 2.3.3 Race Model Violation

RTs to recurrent AV targets are often faster than the ones to the separate components, a phenomenon known as the redundant signals effect (RSE) (Hershenson 1962; Kinchla 1974). The RSE can be explained by parallel processing models, such as the race model, which predicts that a response is triggered independently by the faster sensory modality (Raab 1962). However, the observed RSE typically exceeds the advantage predicted by mere statistical facilitation (Miller 1982). To test the model's temporal detection ability, we computed Cumulative Distribution Functions (CDFs) of RT distributions per condition (A, V, and AV). We quantified the number of AV RTs that were shorter than the quicker unisensory RTs, thereby obtaining the multisensory gain. Simulated benefits were measured as the graphical surface between the CDF of the AV condition and that of the faster among the A or V condition. Assuming statistical independence between the sensory modalities, we used Raab's independent race model (Raab 1962) to derive predicted benefits, quantified as the area between the CDF of the race model and the faster modality‐specific condition.

### 2.4 Sensitivity Analyses

To explore the neural mechanisms that likely underlie modifications of modality switch effects along the spatial coordinate, we performed a sensitivity analysis by systematically varying the effectiveness of two connection types—cross‐modal connections and lateral intra‐area connections within the unisensory areas. These connections were chosen for these analyses because they allow us to reproduce localization tasks and thus primarily influence the performance of the network when the spatial configuration of sensory stimuli changes, as previously demonstrated by Cuppini et al. (2017). Because the switch cost is typically observed in unisensory trials, we simulated RTs to sequential visual or auditory modality‐specific stimuli at ISI randomly varying between 1000 and 3000 ms and computed the switch cost for each unisensory modality (A and V). Results were averaged over 100 simulations for each sensory modality and then over modalities to obtain a single value of switch cost. We repeated this procedure for increasing values of effectiveness of the cross‐modal connections (i.e., W_va0_ = 0.08, 0.012, 0.016) and for increasing values of effectiveness of the inhibitory component of the lateral intra‐area connections within the unisensory areas (i.e., L_in0_ = 3, 4, 5), while maintaining a constant value of the ratio between the effectiveness of excitation and inhibition (i.e., L_ex0_/L_in0_). The results of these analyses are presented and discussed in the Supporting Information.

### 2.5 Predictions in the Spatiotemporal Domain

Predictive simulations in untested conditions were obtained by varying the spatial and temporal coordinates together. As RTs to Rp trials are less sensitive to spatiotemporal variations, we chose to collect RTs to Sw trials (SwV and SwA) for values of ISI from 0 to 1000 ms with a 100‐ms step and from 1000 to 300 ms with a 250‐ms step, for each value of AV Disparity between 0° and 20°, with a 5° step. Sw RTs results for each condition were averaged over 100 simulations. The novelty introduced by these predictions is twofold: (1) they offer original insight on a plausible evolution of modality switch effects along the spatial coordinate and (2) they extend the temporal analysis introduced by Cuppini et al. (2020) to values of ISI closer to and within the temporal window of integration. In a second set of predictive simulations, the auditory perception bias was collected for increasing values of ISI (200 to 800 ms) for values of AV Disparity between 0° and 20°, with a 5° step, to explore the influence of cross‐modal temporal misalignment on the ventriloquist effect. The results were averaged over 250 simulations and compared with data from Wallace et al. (2004).

## 3 Results

### 3.1 Mean RT: Model vs. Data

**FIGURE 2:**
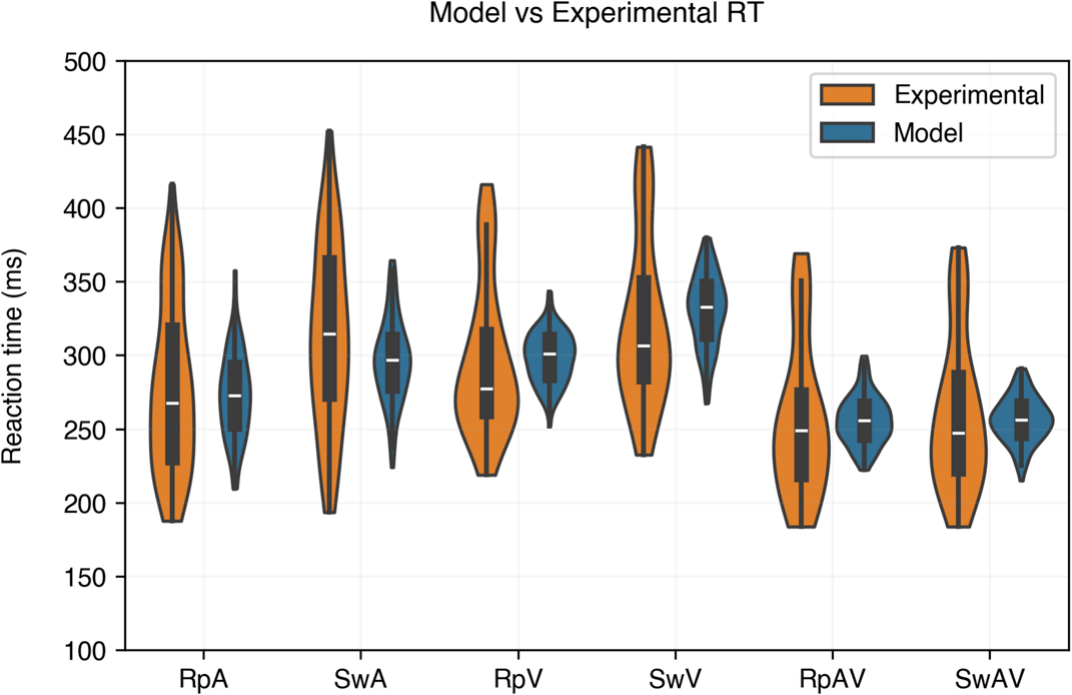
Model vs mean subject RTs. Mean RTs in stimuli configuration Repeat A (RpA), Switch A (SwA), Repeat V (RpV), Switch V (SwV), Repeat AV (RpAV), and Switch AV (SwAV), as explained in the Materials and Methods section: Assessment of network performance. Orange violin plots represent empirical results from Crosse et al. (2022), blue violin plots represent results from model simulations in the corresponding stimuli configuration.

Figure 2 shows that the model in its basal configuration provides a good simulation of the empirical data from adults in Crosse et al. (2022). Results from the three‐way ANOVA revealed a main effect of condition (Rp, Sw—*F*_1/828_ = 53.91, *p* < 0.001) and of modality (A, V, AV—*F*_2/828_ = 46.75, *p* < 0.001), but not of source (empirical data—Crosse et al. 2022, simulated data—Cuppini et al. 2020, simulated data—present model—*F*_*2*/828_ = 0.3327, *p* > 0.05), also confirming the ability of the present model to reproduce the temporal behavior of the model presented in Cuppini et al. (2020). The present model also produces longer RTs in the Sw than in the Rp condition, for both unisensory modalities. In the case of multisensory stimulation, instead, model simulations yield faster RTs, since the effect of cross‐sensory inhibition is attenuated by the excitatory cross‐modal contribution, as well as by the greater activation of the multisensory area. Hence, multisensory RTs do not differ considerably in Sw or Rp trials, contrarily to the unisensory stimulation, and the modality switch effect is not observed.

### 3.2 Race Model Violation

**FIGURE 3:**
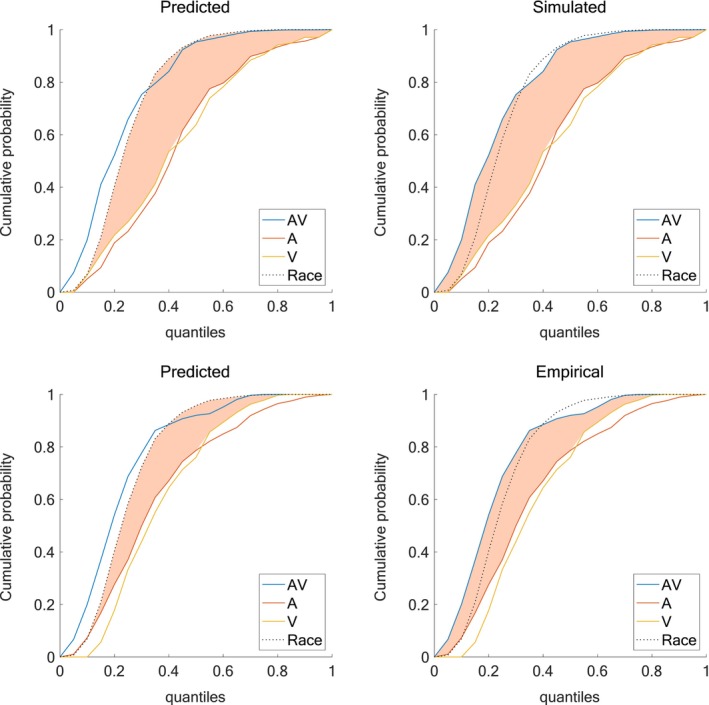
RT cumulative probability for each of the three stimulus conditions and the race model. The top row refers to model RTs; predicted benefits (upper left panel) are quantified by the area between the CDFs of the race model and the faster of the unisensory conditions, while simulated benefits (upper right panel) are quantified by the area between the CDFs of the multisensory condition and the faster of the unisensory conditions. The bottom row refers to empirical RTs; predicted benefits (lower left panel) are quantified by the area between the CDFs of the race model and the faster of the unisensory conditions, while empirical benefits (lower right panel) are quantified by the area between the CDFs of the multisensory condition and the faster of the unisensory conditions. Data from an example NT adult participant.

**FIGURE 4:**
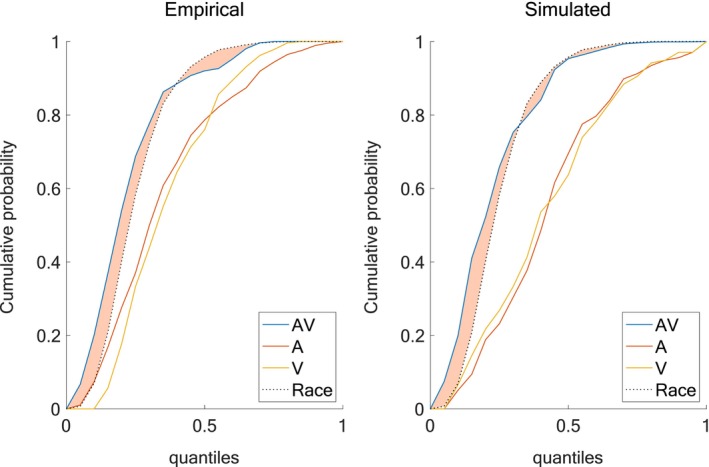
Multisensory gain—AV vs the race model. Left panel refers to data from Crosse et al. (2022) (data from an example NT adult participant), right panel refers to simulated RTs from our model. Multisensory gain is quantified by the area between the CDFs of the multisensory condition and the race model.

To evaluate the extent to which our model captures multisensory facilitation, we compared CDFs of RT quantiles across A, V, and AV conditions, as shown in Figure 3. The predicted data (top‐left and bottom‐left panels) were derived from the race model, which estimates the probability that either unisensory channel produces a response independently. The simulated data (top‐right) were generated from our computational model, and the empirical data (bottom‐right) represent observed participant behavior. In both the simulated and empirical panels, AV RT distributions exceed the race model prediction (dashed line), particularly in the faster quantiles, indicating a violation of race model bounds and thus evidence of multisensory integration. The shaded region between the AV CDF and the race model prediction highlights this violation. Notably, the simulated data closely resemble the empirical distributions across all conditions, capturing both the facilitative effects in the AV condition and the relative ordering of unisensory responses (A faster than V). Therefore, the model is both able to reproduce the central tendency of RTs and the distributional structure of multisensory and unisensory responses. To further assess the model's ability to capture the dynamics of multisensory gain, we plotted CDFs for all sensory conditions in both empirical and simulated datasets (Figure 4). The shaded area between the AV CDF and the most efficient unisensory channel represents the multisensory gain, quantifying the extent to which multisensory responses exceed those predicted by the faster unisensory modality alone. Both panels reveal a robust enhancement in the AV condition, particularly at intermediate quantiles. The shaded regions are similar across empirical and simulated data in shape and extent, further supporting the model's fidelity in reproducing the full profile of multisensory benefit. Besides providing a continuous measure of facilitation across the entire RT distribution, these results suggest that the model also captures the graded nature of multisensory enhancement.

### 3.3 Mean Auditory Perception Bias: Model vs. Data

**FIGURE 5:**
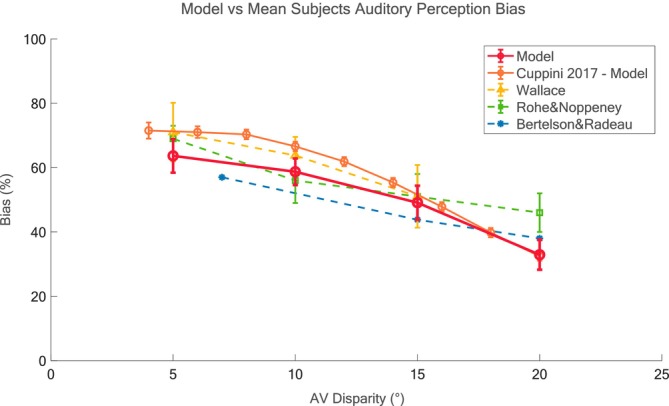
Auditory perception bias (%): Model vs mean subjects data. This index was computed as the difference between the perceived spatial position and the true position of the auditory input, expressed as a percentage of the audiovisual distance. The bias is here represented by mean ± SEM, computed over 100 repetitions for each stimulus configuration. Results from simulations with the same spatial eccentricity to the left and to right were averaged. The results of model simulations (red lines) are compared with Cuppini et al. (2017) (orange lines), and behavioral data present in the literature (yellow lines— Wallace 2004; green lines—Rohe and Noppeney 2015a; blue lines‐Bertelson and Radeau 1981).

As expected (see Figure 5), the auditory bias predicted by the model appears quite uniform for lower values of AV Disparity (5° to 10°), then progressively decreases as AV disparity increases (from a maximum value of about 65% to a minimum of about 35%). A similar pattern is shown by our previous model (Cuppini et al. 2017) as well as the empirical results by Bertelson and Radeau (1981), Wallace et al. (2004), and Rohe and Noppeney (2015a). However, the decrease in bias for higher values of AV Disparity seems to be less pronounced for the empirical data from Rohe and Noppeney (2015a), as in their experiment visual stimulus reliability was modified along with spatial disparity, and we chose to compare our results to those that were more similar to our configurations. It is worth noting that the standard error of the mean (SEM) associated with the present model (red line) is larger than that of the earlier model (orange line), likely due to the increased architectural complexity of the current model, which may amplify the propagation of the added input noise to simulate multiple trials and lead to greater output dispersion.

### 3.4 Model Predictions

**FIGURE 6:**
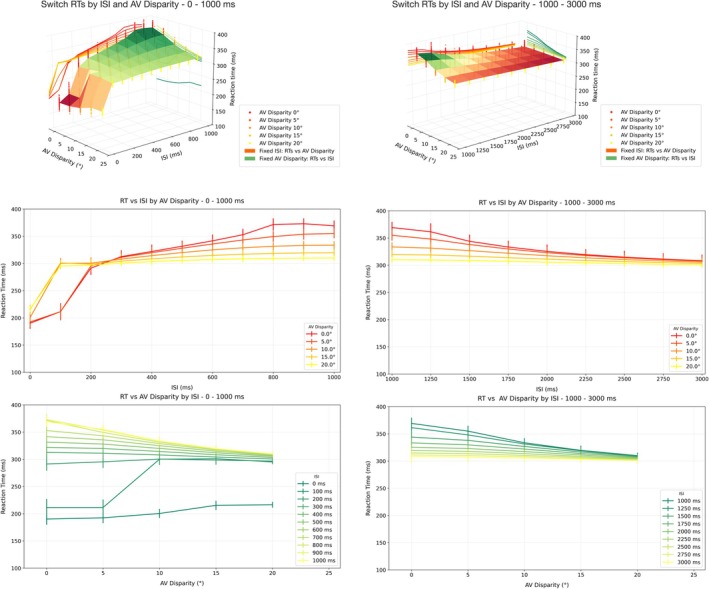
Model predictions—time domain. RTs to switch trials as a function of AV disparity and ISI. Top row: 3D surface plots of mean RTs as a function of AV disparity (x‐axis) and ISI (y‐axis), with data distributions scatterplots for each configuration (yellow to red scatterplot colormap), separated into two temporal windows: early interval (0–1000 ms) (left) and late interval (1000–3000 ms) (right). 2D projections of RTs for fixed ISI are reported on the xz‐plane (orange colormap), while 2D projections of RTs for fixed AV Disparity are reported on the yz‐plane (green colormap). **Middle row:** 2D line plots of RT vs. ISI, grouped by AV disparity levels. **Left (0–1000 ms):** RTs increase with longer ISIs, especially for small disparities (0°–5°), while large disparities (15°–20°) yield slower and more stable responses across ISIs. **Right (1000–3000 ms):** RTs decrease with increasing ISI, particularly for low disparity conditions, indicating a reversal in temporal dynamics across the two windows. **Bottom row:** 2D line plots of RT vs. AV disparity, grouped by ISI levels. **Left (0–1000 ms):** Larger AV disparities lead to greater RTs at short ISIs, while as ISI increases RTs to spatially congruent stimuli become greater than those for spatially discrepant stimuli. **Right (1000–3000 ms):** RTs decrease over increasing AV disparity and ISI.

Figure 6 illustrates the effects of ISI and AV disparity on Sw RTs across two distinct time windows: 0–1000 ms (left panels) and 1000–3000 ms (right panels). The top row presents 3D surface plots of RTs as a function of ISI and AV disparity for each temporal segment, with scatterplots of data distributions for each configuration. 2D projections of RTs are also depicted, both in function of ISI only for different values of AV Disparity, reported in the Figure 6 second row, and in function of AV Disparity only for different values of ISI, reported in the Figure 6 third row. As expected, in the 0–1000‐ms window, RTs increase with increasing AV disparity and ISI, particularly for disparities up to 15°, while the 1000–3000‐ms window exhibits an overall decrease in RTs with longer ISIs and smaller AV disparities. Line plots in the middle row show RTs as a function of ISI for fixed AV disparities. In the early window (0–1000 ms), RTs increase monotonically with ISI, with a steeper rate of increase for low AV disparities (0°–5°) compared to higher disparities (15°–20°), where the trend is more gradual. In contrast, in the later window (1000–3000 ms), RTs decrease with increasing ISI, and this effect appears more pronounced at low AV disparities. The bottom row displays RTs as a function of AV disparity at fixed ISIs. For 0–1000 ms, RTs show different evolutions based on ISI: At 0‐ms ISI, RTs appear quite rapid in condition of spatial congruency or quasi‐congruency (0°–5°) and increase as the disparity between the cues increases. The same trend is visible for curves relative to ISIs within or near the temporal window of integration (100–200‐ms ISI), so that AV stimuli presented in spatial congruency or quasi congruency (0°–5°) are detected markedly faster than those delivered in spatial disparity (10° to 20°). Though steeper for 100‐ms ISI, the increase in RT between 5° and 10° is visible at 200° too.

On the other hand, a completely different trend emerges for ISI ranging between 300 and 1000 ms, where for 300–500‐ms ISI, RTs generally stabilize over all AV disparities, to exhibit then maximal values at lower disparities and decrease along the spatial coordinate in the ISI range between 600‐ and 1000‐ms ISI. This opposite pattern is likely due to the emerging effect of cross‐sensory competition, peaking around 1000 ms, which is maximum at lower disparities and becomes attenuated as the component stimuli are separated in space.

Consistently, in the 1000–3000‐ms interval, RTs show a decrease with AV disparity across all ISIs, though the magnitude of this effect diminished as ISI increases.

The results of the last set of predictive simulations are reported in Figure 6. The predicted auditory bias in the four conditions linearly decreases as the ISI increases. For ISIs of 200 and 500 ms, the bias appears higher for all values of spatial disparity reflecting an enhanced tendency for visual attraction, whereas sensible improvements in auditory localization are observed for longer ISI (=800 ms). A similar pattern is shown by Wallace's data, whose values of SEM (for all points) appear to be slightly higher than ours and allow us to state that our results fall within the experimental data distribution. Notably, the curve associated with the 20° disparity represents a novel prediction whose decreasing trend, although requiring further empirical validation, aligns reasonably well with those observed for lower disparities.

## 4 Discussion

Spatiotemporal sensory perception is perhaps one of the most complex issues that the neuroscientific community is currently reflecting upon, remarkably difficult to approach from an experimental point of view. Therefore, a large body of computational models has been proposed that successfully accounted for various kinds of temporal detection tasks, or could better explain the brain's behavior in spatial localization. Unfortunately, quite fewer have tried to reunite these two aspects of multisensory perception. An example is provided by the nonhierarchical variant of the classical Bayesian causal inference model (Shams et al. 2005), which was proposed by Körding et al. (2007) to reproduce data from AV spatial localization tasks. Later, the same model was also tested in temporal numerosity tasks and showed to account for the data very well in bisensory conditions (Odegaard and Shams 2016). While many models of this kind have provided important insights into the computational strategies that may underlie multisensory perception (see Shams and Beierholm 2022 for a review), the experimental efforts aimed at mapping these computations to their plausible neural correlates are recent. For example, the posterior parietal cortex and superior temporal sulcus have been suggested to represent both sensory uncertainty and causal structure (Aller and Noppeney 2019; Ferrari and Noppeney 2021; French and DeAngelis 2020; Rohe and Noppeney 2015a). At the same time, studies investigating the neural encoding of probability distributions have found that population‐level activity in sensory cortices reflects sensory uncertainty and prior expectations (Fetsch et al. 2012; Fetsch et al. 2013; Van Bergen et al. 2015).

An interesting work by Sato et al. 2007 also proposes a computational approach to AV integration, describing a Bayesian observer that estimates the location and timing of visual and auditory stimuli using a maximum a posteriori approach, incorporating spatial and temporal sensory noise. Rather than assuming that AV targets should be bound, the model infers source unity probabilistically, allowing for flexible judgments based on prior beliefs. The model successfully captures several key perceptual phenomena, including the ventriloquism effect, its association with spatial unity perception, and the mutual influence of spatial and temporal discrepancies on cross‐modal judgments. From the same group, Terada et al. 2024 show how chaotic dynamics in recurrent neural networks can support Bayesian inference through sampling. Variability emerging from deterministic chaos enables the network in this work to approximate posterior distributions in cue integration tasks. The focus of this study mainly remains at the computational level, but connections are drawn to neural circuitry and align with experimental observations of irregular brain activity. Another approach was proposed by Lerousseau et al. 2022, based on single computational units working as multisensory correlation detectors. Each unit filters single sensory signals and then combines them linearly to detect correlation and time lag, thus performing temporal order judgments. An ensemble of detectors then integrates cues optimally in space. Compared with our model, Lerousseau and colleagues realize integration completely in a multisensory layer (as units are intrinsically multisensory) and include a time lag detector explicitly, while it is the overall behavior of our network that computes integration or segregation. Moreover, our model incorporates lateral inhibitory mechanisms and nonlinear saturation, which play an important role in multisensory interactions (see Ursino et al. 2014).

Alternatively, Oess et al. (2020) developed a physiology‐based neural network model featuring multisensory neurons that receive excitatory and inhibitory inputs from unisensory auditory and visual neurons, as well as feedback from the cortex. The model is proposed in two versions, i.e., a rate‐based model for qualitative analysis and a variant with spiking neurons that has been implemented on a neuromorphic processing system. Tested with audiovisual cues of different intensities and spatial offsets, the network proved capable of near‐optimal Bayesian integration of cross‐modal stimuli. While this work is primarily oriented towards detailing the computational strategies of the neural circuits subtended to integration, our model has been mainly conceived to investigate multisensoriality in behavioral tasks and thus tested in spatial but also temporal varying conditions. Nonetheless, both models can be considered biologically plausible, as they are largely inspired by prior neurophysiological findings.

The aim of the model described in this work is twofold: (1) to reproduce experimental results for tasks of sensory perception, in case of manipulation of the temporal (Crosse et al. 2022) and spatial properties (Bertelson and Radeau 1981; Rohe and Noppeney 2015b; Wallace et al. 2004) of the external sensory stimuli; and (2) to offer a valid theoretical framework for the analysis of multisensory phenomena in the entire spatiotemporal domain and for the generation of new testable predictions on the mechanisms regulating sensory perception in a complex environment. To this purpose, we started from the model presented in Cuppini et al. 2020, developed for temporal investigations, and we introduced several modifications that would allow the network to also reproduce space‐oriented experiments. A novelty of the present work is the delivery of AV stimuli at multiple spatial positions, since we considered 180 neural units in each area instead of a single neural element (as in Cuppini et al. 2020). In the unisensory areas, each of the neural units now codes for a specific location of the external space and exchanges distributed connections with the corresponding unit in the other unisensory area. Plenty of evidence in literature confirms the existence of cross‐modal links between the primary visual and auditory regions (Alais et al. 2010; Foxe and Schroeder 2005; Ghazanfar and Schroeder 2006; Musacchia and Schroeder 2009; Recanzone 2009; Shams and Kim 2010), and that visual inputs to auditory neurons exert subthreshold influences, able to modulate the responses to sound (Bizley and King 2009). In accordance with these data, previous modelling works demonstrated that cross‐modal excitatory connections explain popular illusory phenomena, such as spatial ventriloquism (Cuppini et al. 2017; Magosso et al. 2012) and the sound‐induced flash illusion (Cuppini et al. 2014). Other studies by Campus et al. (2017) and Gori (2015) showed that an early response of an extended area of the temporal cortex, likely including the auditory area, can be elicited by presenting visual stimuli in a temporal bisection task, while spatial localization tasks of sounds can evoke early responses in the occipital cortex, with task‐specific amplifications that are linked to the position of sound in the two hemifields (Amadeo et al. 2020).

An additional facilitatory mechanism in the model is implemented by the feedforward synapses converging from the auditory and visual areas towards the multisensory area, where the component unisensory signals are integrated and further feedforward synapses can activate the premotor area to elicit a motor response. The presence of a sigmoidal relationship in the multisensory region is crucial to explain some key principles of multisensory integration, such as enhancement and inverse effectiveness (Cuppini et al. 2012; Cuppini et al. 2018; Magosso et al. 2008). In other words, feedforward and cross‐modal connections are responsible for recreating the canonical pattern of multisensory integration, and it is worth noting that they have been implemented with a small synaptic time constant (15 ms), of the same order as the one used to mimic the auditory response. Hence, these mechanisms truly function to integrate auditory and visual stimuli in close temporal proximity, as predicted by several studies on the temporal window of integration (Stein and Meredith 1993; Miller et al. 2015; Rowland et al. 2007).

An alternative explanation for the speedup in the RTs observed for AV stimuli might be that subjects are merely responding to the first stimulus detected. According to the race model, the observed RT is representable by the faster visual or auditory signal leading to a purely statistical facilitation effect in response speed. In accordance with previous studies (Diederich and Colonius 2004b; Miller 1982), the race model is not sufficient to explain the multisensory RTs simulated by our model, as well as empirical RTs from Crosse et al. (2022). Our analyses on the simulated RSE indeed proved the robustness of our network against the race model hypothesis, confirming that the RT to a combined AV stimulus on any trial does not correspond to the faster modality on that trial. Along with cross‐modal connectivity, also intra‐area lateral projections implementing competition within the same layer play a central role in explaining the spatial behavior of the model. These synapses are organized in the shape of a Mexican hat, with stronger effectiveness among neural elements in the unisensory areas to allow an amelioration of spatial perception. When a cross‐modal stimulus is delivered to the input layer, these connections promote the formation of an activation bubble in each unisensory area, close to the true position of the visual component in the visual area (pv), and of the acoustic component in the auditory area (pa). The presence of noise may affect the bubble position, especially for the auditory input which is less effective and less resolved in space. At this point, Gaussian‐shaped cross‐modal connections sustain progressively stronger feedback between the two activation bubbles, with the visual bubble exerting a greater impact on the auditory one due to sharper connectivity from the visual pathway. Consequently, the auditory activity in proximity of the spatial position pv increases, and this activation competes due to lateral inhibition with the original auditory activity at position pa. Eventually, the auditory activation at position pv becomes predominant, sensibly attenuating the auditory activation at position pa and the surrounding positions, and ultimately revealing the localization bias that characterizes the ventriloquist effect.

A further perceptual phenomenon the model aims to account for, and to expand upon, is the modality switch effect. A feedback competitive loop mediated by inhibitory synapses between the two sensory modalities is responsible for reproducing switch costs thanks to a long‐lasting inhibition activated by the first stimulus over the second. This process has a much longer dynamics than those of multisensory integration and operates with a time scale compatible with the RTs observed by Crosse et al. (2022). Interestingly, this inhibitory mechanism plays a relevant role only in the presence of sequential unisensory stimuli of different sensory modalities. It is known to be stronger for stimuli separated by a 1000‐ms interval, and it decreases with the ISI, showing almost no effect for stimuli separated by a 3000‐ms interval. Nonetheless, a crucial aim of this model was to generate novel predictions on the modulation of perceptual performance by the spatiotemporal relationships between AV targets. In the time domain, RTs to switch trials were collected because of their particular sensitivity to spatiotemporal offsets variations, while systematically varying the ISI between the stimuli from 0 to 3000 ms, and the AV Disparity between the stimuli from 0° to 20°. In the space domain, the auditory perception bias values were collected with AV Disparity varying between 0° and 20°, and ISI varying between 200 and 800 ms to compare them with results from Wallace et al. (2004). In the temporal domain, Sw RT predictions show different trends for different intervals of ISI values (Figure 6). First, the model generates the fastest RTs in response to cross‐modal stimuli delivered synchronously or within the temporal binding window (0–100‐ms ISI) and in spatial congruency (0°–5°), consistent with the spatial and temporal rule regulating multisensory integration at the neural as well as the behavioral level (Stein 2012). Additionally, an increase in spatial disparity, even in conditions of temporal synchrony, produces a visible slowdown in RTs, which is confirmed by previous results on the negative effect of cross‐modal spatial disparity on behavioral performance (Frens et al. 1995; Stevenson et al. 2012). Even though to a lesser extent, also at 200‐ms ISI RTs resulted faster at a lower spatial disparity and show a slight increase along the spatial coordinate, suggesting that the temporal window of this model lies indeed between 0 and 200 ms and is consistent with behavioral reports of 200–400 ms as upper limits for temporal integration (Zhou et al. 2020). From 300 ms towards 600‐ms ISI, RTs seem to stabilize at constant values across AV Disparities, suggesting that the loss of temporal synchrony may remove the behavioral benefit associated with integration at null or low disparity. Noteworthily, these predictive simulations reveal a transitional phase between integration and competition. Empirical data on this interval are relatively sparse, due to experimental limitations associated with short ISIs, where subjects may struggle to determine which stimulus to respond to or whether to integrate them. Our model provides a computationally precise account of the underlying dynamics, showing that in this regime cross‐modal interactions are neither fully integrative nor entirely competitive. Rather, the system appears to be in a metastable state that may represent a critical window for shaping the temporal boundaries of perceptual binding and the onset of cross‐sensory inhibition, offering an important target for future psychophysical and neurophysiological investigation. Moving to 700‐ to 1000‐ms ISI, unisensory stimuli cannot be considered components of a cross‐modal stimulus anymore; rather, they become sequential modality‐specific stimuli. The dynamics of cross‐sensory inhibition become increasingly effective, and the predicted evolution of RTs along the spatial coordinate is reversed, so that RTs are slower at smaller AV Disparities and decrease as the disparity between the stimuli increases. Faster RTs to spatially disparate sensory targets delivered sequentially in time have also been observed in a number of studies investigating the phenomenon known as inhibition of return, which refers to the delayed detection often found for targets at the same location as a preceding event (Klein 2000). In the multisensory domain, inhibition of return has been shown to extend across‐modalities (e.g., Spence et al. 2000; Tassinari and Campara 1996), such that a stimulus presented in one modality can inhibit the response to a subsequent stimulus presented at the same location but in a different modality, supporting the notion that spatial separation between temporal offsets between sensory cues may serve to reduce cross‐modal interference and facilitate faster detection. Critically, recent empirical findings from Odegaard et al. 2017 provided additional behavioral evidence suggesting that temporally correlated but spatially discrepant stimuli can enhance perceptual binding performance, so that a finite spatial disparity may be beneficial under conditions of temporal conflict, potentially allowing the system to disambiguate the sensory sources and resolve competition between modalities. This is particularly relevant in contexts where Modality Switch effects emerge, as spatial separation may attenuate the inhibitory influence of the preceding modality and support faster engagement of modality‐specific processing pathways. The second set of predictions aimed at observing changes in auditory perception bias for increasing values of ISI and AV spatial disparity. As expected, both model simulations and data from Wallace et al. (2004) indicate that increasing temporal discrepancy between the auditory and visual cues considerably reduces the effect of visual dominance on audition, with a similar trend to that observed for increasing spatial disparity between synchronous stimuli (reported in Figure 3). These findings agree with previous reports where both spatial and temporal separations induced improvements in auditory perceptual accuracy (Lewald et al. 2001; Slutsky and Recanzone 2001).

**FIGURE 7:**
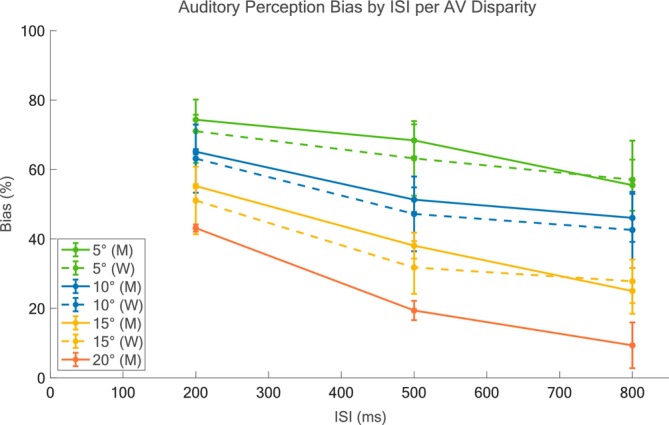
Model predictions—Space domain. Auditory Perception Bias as a function of ISI for multiple values of AV disparity. Auditory Perception Bias values from model simulations are represented by mean ± SEM, as a function of ISI, for different values of AV Disparity (indicated with M, 5°—green solid line, 10°—blue solid line, and 15°—yellow solid line, 20°—orange solid line). Results for each value of spatial disparity are then compared with the corresponding experimental results from Wallace 2004 (indicated with W, 5°—green dotted line, 10°—blue dotted line, and 15—yellow dotted line). For each value of the AV distance, biases were averaged over 250 repetitions, and then the mean bias obtained for equal values of spatial disparity to left and to the right were averaged with one another.

Closely related to the issue of the stimuli localization is the source estimation process, or the causal inference problem (Fetsch et al. 2013; Jones and Noppeney 2024; Shams and Beierholm 2022). For instance, the auditory perception bias has been found to change sensibly in the case two stimuli are considered to originate from a common cause, or from separate sources (see Aston et al. 2023 for a review), and depending on the sensory experience with multisensory events (Monti et al. 2023). Although the present model does not explicitly simulate the source estimation process central to Bayesian Causal Inference frameworks, the observed auditory localization biases (Figure 4) and the spatial predictions from the current model (Figure 7) are consistent with the modulation of perception based on the inferred likelihood of a common source (Odegaard et al. 2015; Rohe and Noppeney 2015b). Future extensions could directly incorporate causal inference mechanisms to examine how spatiotemporal disparities between sensory cues, such as variations in onset asynchrony or spatial offset, affect source estimation.

### 4.1 Model Limitations and Future Lines

Though able to account for diverse multisensory phenomena, the present model has some limitations. First, we assumed that stimuli reliability is independent of the azimuthal coordinate position. Since we chose to simulate AV disparities no greater than 20°, we believe that introducing larger receptive fields for peripheral vs. central neurons would have increased the complexity of the model excessively.

We are also aware that noise in real biological networks can originate at different perceptual levels, beyond the effectiveness of the input stimulus which we manipulated in this work to simulate within‐subject variations. It would be interesting to introduce noise in the main synaptic mechanisms acting in the network by systematically simulating a change in both their effectiveness and diffusion, and in this way simulate empirical intersubject variations that are observed in the two tasks (RT and spatial localization).

Furthermore, these results demonstrated that the mechanisms implemented in the unisensory layers exert a major role in generating sensory perception, but we are aware that real biological networks include feedback projections from higher order to primary cortical regions. Future experiments will be conducted to analyze the role of these projections in regulating spatiotemporal sensory processing.

Finally, we mimicked localization in the auditory area in the same way as in the visual, despite such topological organization being typical of the visual cortex only. Nonetheless, physiological evidence shows that a spatial map of sound in which neurons are unambiguously tuned to specific sound locations is present in the superior colliculus, a critical structure for reflexive orienting movements of the head and eyes. The auditory area in our model aims at summarizing a multiplicity of structures involved in the distributed neural representation of sounds (see Kandel 2013 for completeness) that synergistically process binaural and monaural spectral cues to determine the azimuthal and vertical position of sound and guide orientation as elicited in the examined behavioral tasks.

## 5 Conclusion

Our work provides unprecedented preliminary insights on the spatial modulation of intersensory switching, which significantly influences response times and perceptual accuracy in behavioral experiments. By demonstrating how spatial proximity and disparity modulate audiovisual detection, our novel predictions point to the relevance of carefully controlling the spatial and temporal alignment of stimuli in psychophysical testing, as precise tuning of spatiotemporal factors can reduce ambiguity and lead to an amelioration in the recorded perceptual performance. These findings are particularly relevant for creating robust behavioral tasks that can account for inter‐individual differences and the dynamic interplay of multisensory integration.

## Author Contributions

**Cristiano Cuppini:** conceptualization, funding acquisition, investigation, methodology, project administration, resources, software, supervision, writing – review and editing. **Eleonore F. Di Rosa:** data curation, formal analysis, investigation, software, visualization, writing – original draft. **Laura Astolfi:** funding acquisition, resources, supervision, writing – review and editing. **Melissa Monti:** conceptualization, investigation, methodology, software, supervision, writing – review and editing.

## Conflicts of Interest

The authors declare no conflicts of interest.

## Peer Review

The peer review history for this article is available at https://www.webofscience.com/api/gateway/wos/peer‐review/10.1111/ejn.70217.

## Supporting information

Supplementary Material.

## Data Availability

Data will be made available upon request.
