# Supplementary material for: Unraveling Audiovisual Perception Across Space and Time: A Neuroinspired Computational Architecture
Source: Eur J Neurosci. 2025 Aug 5;62(3):e70217. doi: 10.1111/ejn.70217 (PMC12326128; doi:10.1111/ejn.70217)
Supplement: Supplementary file 1 — Supplementary Material. [file EJN-62-0-s001.docx]

Supplementary material

1.1 Mathematical description of the model

In the following, each element will be indicated with a superscript, r (r = a, v, m, pm, ia, or iv, where a refers to the auditory input area, v to the visual input area, m to the multisensory/motor output region, pm to the premotor area, ia and iv to the auditory and visual interneurons, respectively), and subscript j, which denotes the spatial position within that area. Thus, $u_{j}^{r}\left( t \right)$ and $y_{j}^{r}\left( t \right)$ represent the net input and output, respectively, of a neural unit in position j, belonging to area r, at time t. The output $y_{j}^{r}\left( t \right)$ is described by the following differential equation:

$\tau^{r}\frac{dy_{j}^{r}(t)}{dt}= -y_{j}^{r}\left( t \right)+F(u_{j}^{r}\left( t \right))$ (1)

where τ is the time constant and F(u) represents the sigmoidal relationship:

$F\left( u_{j}^{r} \right)=\frac{1}{1+e^{-s(u_{j}^{r}-\theta)}}$ (2)

s and θ establish the slope and the central position of the sigmoidal relationship, respectively. The net input entering a specific neural element (i.e. the quantity $u_{j}^{r}\left( t \right)$ in Eq. 1) depends on the area it resides in:

***Input areas*** - The input to the elements in these areas is the made up of two terms: (1) the contribution of lateral synapses from other units in the same area ($l_{j}^{r}\left( t \right)$), and (2) a component coming from outside the area ($i_{j}^{r}\left( t \right)$). Hence:

$u_{j}^{r}\left( t \right)= l_{j}^{r}\left( t \right)+ i_{j}^{r}\left( t \right)$ (3)

To simulate the lateral input, $l_{j}^{r}\left( t \right)$, each unit within the unisensory areas excites (and is excited by) adjacent units, and inhibits (and is inhibited by) more distal units. Thus, the input that any unit receives from other elements of the same area is:

$l_{j}^{r}\left( t \right)= \sum_{k} L_{jk}^{r}\cdot y_{k}^{r}\left( t \right)$ (4)

where $L_{jk}^{r}$ is the effectiveness of the lateral synapse from a presynaptic unit at position k to a postsynaptic unit at position j both belonging to the same region r, and $y_{k}^{r}\left( t \right)$ is the activity of the presynaptic unit at position k. These synapses are symmetrical and arranged in a “Mexican hat” distribution (a central excitatory zone surrounded by an inhibitory crown):

$L_{jk}^{r}=\left\{ \begin{aligned} L_{ex0}^{r}\cdot e^{-\frac{\left( d_{jk} \right)^{2}}{2\left( \sigma_{ex}^{r} \right)^{2}}}-L_{in0}^{r}\cdot e^{-\frac{\left( d_{jk} \right)^{2}}{2\left( \sigma_{in}^{r} \right)^{2}}}, if d_{jk} \neq0 \\ 0, if d_{jk} =0 \end{aligned} \right.$ (5)

where $L_{ex0}^{r}$ and $\sigma_{ex}^{r}$ define the excitatory Gaussian function, $L_{in0}^{r}$ and $\sigma_{in}^{r}$the inhibitory one, and $d_{jk}$ is the distance between the pre and post-synaptic unit in the same area. To avoid border effects, we used a circular synaptic structure so that every unit in each area receives the same number of side connections. This is obtained applying the following expression for the distance:

$d_{jk}=\left\{ \begin{aligned} \left| j-k \right|, if \left| j-k \right| \leq\frac{N}{2} \\ N- \left| j-k \right|, if \left| j-k \right| >\frac{N}{2} \end{aligned} \right.$ (6)

To limit the number of hypotheses, we assumed that both unisensory areas have the same pattern of lateral synapses.

The contribution of extra-area sources, $i_{j}^{r}\left( t \right)$, is the sum of three terms:

$i_{j}^{r}\left( t \right)= e_{j}^{r}\left( t \right)+ c_{j}^{r}\left( t \right)+ {Li}_{j}^{r}\left( t \right), r=a, v$ (7)

The external input $e_{j}^{r}\left( t \right)$ is simulated with a spatial Gaussian distribution, to reproduce the uncertainty in its detection. Assuming a stimulus of sensory modality r (r = a or v), with a duration $T^{r}$, is presented in the position $p^{r}$, the input to the network results:

$e_{j}^{r}(t)=\left\{ \begin{aligned} E_{0}^{r}\cdot e^{-\frac{\left( d_{j}^{r} \right)^{2}}{2\left( \sigma^{r} \right)^{2}}}, if 0 \leq t\leq T^{r} \\ 0, if t> T^{r} \end{aligned} \right.$ (8)

where $E_{0}^{r}$ is the strength of the stimulus, $d_{j}^{r}$ the distance between the unit at position j and the stimulus at position $p^{r}$, and $\sigma^{r}$ the degree of uncertainty in detection. The external inputs have a circular structure too, for the same reason cited above.

The cross-modal input, $c_{j}^{r}\left( t \right)$, is computed assuming that each unisensory unit is excited by the units processing the other modality. Hence

$c_{j}^{a}\left( t \right)= \sum_{k=1}^{N} W_{jk}^{av}\cdot y_{k}^{v}(t- \Delta t)$ (9)

$c_{j}^{v}\left( t \right)= \sum_{k=1}^{N} W_{jk}^{va}\cdot y_{k}^{a}(t- \Delta t)$ (10)

Where $\Delta t$ is the latency in the exchange of cross-modal inputs between the two areas, and $W_{ij}^{va}$, $W_{ij}^{av}$, are Gaussian distributed, i.e.

$W_{jk}^{cd}= W_{0}^{cd}\cdot e^{-\frac{\left( d_{jk} \right)^{2}}{2\left( \sigma^{cd} \right)^{2}}}, cd=av or va$ (11)

and $W_{0}$ is the maximum level of synaptic efficacy, and $d_{jk}$ is the distance defined similarly to in Eq. 5. $\sigma^{cd}$ is the width of the cross-modal synapses. The pattern of the cross-modal synapses is not symmetrical, in particular $W_{0}^{av}$ is greater than $W_{0}^{va}$, and $\sigma^{av}$ is greater than $\sigma^{va}$ , for the visual processing stream exerts a stronger influence on the auditory area, has greater spatial acuity, especially in auditory localization tasks.

Finally, the inhibitory input ${Li}_{j}^{r}(t)$, generated by the other modality interneuron region, is defined as

${Li}_{j}^{a}\left( t \right)= \sum_{k=1}^{N} {LaIv}_{jk} \cdot y_{k}^{iv}(t)$ (12)

${Li}_{j}^{v}\left( t \right)= \sum_{k=1}^{N} {LvIa}_{jk} \cdot y_{k}^{ia}(t)$ (13)

where $y_{k}^{iv}(t)$ and $y_{k}^{ia}(t)$ convey the activities of the presynaptic auditory and visual interneurons, and ${LaIv}_{jk}$, ${LvIa}_{jk}$ are the strengths of the inhibitory synapses. Again, these synapses are a Gaussian distributed, i.e.

${LaIv}_{jk}= {LaIv}_{0}\cdot e^{-\frac{\left( d_{jk} \right)^{2}}{2\left( \sigma^{aIv} \right)^{2}}}$ (14)

${LvIa}_{jk}= {LvIa}_{0}\cdot e^{-\frac{\left( d_{jk} \right)^{2}}{2\left( \sigma^{vIa} \right)^{2}}}$ (15)

and ${LaIv}_{0}$ and ${LvIa}_{0}$ are the maximum values of synaptic efficacy, $d_{jk}$ is the distance defined similarly to in Eq. 5. $\sigma^{aIv}$ and $\sigma^{vIa}$ is the width of the feedback inhibitory synapses. The pattern of these synapses is not symmetrical, in particular ${LvIa}_{0}$ is greater than ${LaIv}_{0}$, while $\sigma^{aIv}$ = $\sigma^{vIa}$. In the model, we do not incorporate a delay for the cross-sensory inhibition, because the chosen dynamics of the inhibitory effect is noticeably longer than every other mechanism of the network.

***Inhibitory interneural areas*** - Units in these two regions (r = Ia, Iv) are excited respectively by the corresponding units in the auditory and visual areas. They exchange inhibitory connections, implementing a “Winner-Takes-All” (WTA) mechanism. Their net input is the result of an excitatory stimulus, $I_{ex}^{r}(t)$, coming from the corresponding unisensory unit through excitatory synapses; and an inhibitory component, $I_{in}^{r}(t)$, produced by inhibitory synapses from the other interneuron area.

The excitatory components, $I_{ex}^{r}(t)$, targeting the auditory and visual interneurons are defined as:

$I_{exj}^{ia}\left( t \right)= \sum_{k=1}^{N} {WIa}_{jk} \cdot y_{k}^{a}(t)$ (17)

$I_{exj}^{iv}\left( t \right)= \sum_{k=1}^{N} {WIv}_{jk} \cdot y_{k}^{v}(t)$ (18)

where ${WIa}_{jk}$ and ${WIv}_{jk}$ are the weights of the excitatory connections from a unisensory unit to its corresponding interneuron element, and they are described by a Gaussian distribution. Hence,

${WIv}_{jk}= W{Iv}_{0}\cdot e^{-\frac{\left( d_{jk} \right)^{2}}{2\left( \sigma^{Iv} \right)^{2}}}$ (19)

${WIa}_{jk}= W{Ia}_{0}\cdot e^{-\frac{\left( d_{jk} \right)^{2}}{2\left( \sigma^{Ia} \right)^{2}}}$ (20)

where $W{Iv}_{0}$, and $W{Ia}_{0}$ are the highest levels of synaptic efficacy, $d_{jk}$ is the distance between the unit at position j in the post-synaptic interneural area and the unisensory unit at position k in the pre-synaptic area. $\sigma^{Iv}$ and $\sigma^{Ia}$ define the width of the excitatory synapses. The peak synaptic efficacy of these synapses has been set so that $W{Iv}_{0}$ = $W{Ia}_{0}$/ $AV ratio$, where AV ratio = 3/5, while the width is the same for both connections ($\sigma^{Iv}$ = $\sigma^{Ia}$).

The inhibitory input, $I_{in}^{r}(t)$, that an interneural unit receives from the interneural unit of the other modality is defined as:

$I_{inj}^{ia}\left( t \right)= \sum_{k=1}^{N} L_{jk}^{av} \cdot y_{k}^{Iv}(t)$ (21)

$I_{inj}^{iv}\left( t \right)= \sum_{k=1}^{N} L_{jk}^{va} \cdot y_{k}^{Ia}(t)$ (22)

where $y_{k}^{Iv}(t)$ and $y_{k}^{Ia}(t)$ are the activities of the presynaptic auditory and visual interneurons, respectively, and $L_{jk}^{av}$ and $L_{jk}^{va}$ are the weights of the reciprocal inhibitory connections, describing a 1-to-1 connectivity between the units of the interneuron areas. These symmetrical synapses ($L_{jk}^{av}$ = $L_{jk}^{va}$) implement the WTA mechanism between the two areas. Also in this case, as in Eq. 15 and 16, we do not include a pure delay for the same reason stated above.

***Multisensory area*** - this region (r = m) receives a net input that is the sum of the stimuli, carried by long-range excitatory synapses, from the auditory and visual input areas. Hence,

${ex}_{j}^{m}\left( t \right)= \sum_{k=1}^{N} W_{jk}^{ma} \cdot y_{k}^{a}\left( t-{\Delta t}^{m} \right)+\sum_{k=1}^{N} W_{jk}^{mv} \cdot y_{k}^{v}\left( t-{\Delta t}^{m} \right)$ (23)

where ${\Delta t}^{m}$ represents the slightest latency with which stimuli from the input regions are able to generate behavioral responses; $W_{jk}^{ma}$ and $W_{jk}^{mv}$ are the weights of the synapses linking the presynaptic unit at position k in the unisensory area (auditory and visual, respectively) to the j-th unit in the multisensory area. The effectiveness of these synapses is described by a Gaussian distribution, i.e

$W_{jk}^{mc}= W_{0}^{mc}\cdot e^{-\frac{\left( d_{jk} \right)^{2}}{2\left( \sigma^{mc} \right)^{2}}}, c=a,v$ (24)

where $W_{0}^{mc}$ is the highest level of synaptic efficacy, $d_{jk}$ is the distance between the multisensory unit at position j and the unisensory unit at position k, and $\sigma^{mc}$ defines the width of the feedforward synapses. The peak synaptic efficacy of these synapses have been set so that $W_{0}^{mv}= {W_{0}^{ma}}/{AV ratio}$, where $AV ratio=3/5$, while the width is the same for both connections ($\sigma^{ma}$ = $\sigma^{mv}$).

***Premotor area*** - Each unit in this region (r = pm) receive an excitatory input from a unit in the multisensory area in the same position, as they are linked via 1-to-1 connections. Thus, the net input to the premotor area is defined as

$I_{j}^{pmm}(t)= \sum_{k=1}^{N} W_{jk}^{pmm} \cdot y_{k}^{m}\left( t-{\Delta t}^{pm} \right)$ (25)

where $W_{jk}^{pmm}$ are the weights of the synapses between the k-th presynaptic multisensory unit and the j-th postsynaptic premotor unit, and $y_{k}^{m}\left( t \right)$ is the activity of the k-th multisensory neuron. Again, a delay ${\Delta t}^{m}$ is introduced to account for the latency of stimuli from the input regions in generating motor responses.

***Dynamics of each input component*** - All the previous quantities (Eqs (3) to (25)) affect the input $u_{j}^{r}\left( t \right)$ of the corresponding postsynaptic unit via a second order differential equation. By denoting $o_{i}\left( t \right)$ the output of the differential equation for the generic input source $i(t)$ (described by any of Eqs. (3) to (25)) we have

$\left\{ \begin{aligned} \frac{d}{dt}o_{i}\left( t \right)= \delta_{i}\left( t \right); \\ \frac{d}{dt}\delta_{i}\left( t \right)= \frac{G_{i}^{r}}{{(\tau_{i}^{r})}^{2}}i\left( t \right)-\frac{{2\delta}_{i}\left( t \right)}{\tau_{i}^{r}}- \frac{o_{i}\left( t \right)}{{(\tau_{i}^{r})}^{2}} ; \end{aligned} \right.$ (26)

where $G_{i}^{r}$ represents the gain and $\tau_{i}^{r}$ defines the time constant of the dynamics, for each region, r, and input component, i (Eqs. 3 to 25). Eq. 26 implements a second-order impulse response with two coincident real poles. This is used frequently in neural modeling to mimic synaptic dynamics (Cuppini et al., 2014; Jansen & Rit, 1995). To reduce the number of parameters, we choose the same values for $G_{i}^{r}$ and $\tau_{i}^{r}$, for every connection, except for two cases: (1) the external stimuli, and (2) the feedback synapses implementing the cross-sensory inhibitory mechanism. All the equations included in the model were solved numerically in Matlab.

1.2 Sensitivity analyses

**To evaluate the novel contribution of the spatial mechanims introduced in the model, we performed a sensitivity analysis focusing on the cross-modal connections (Wc) and the lateral intra-area connections (Lin). In both analyses, we quantified the switch cost (in milliseconds) as a function of audiovisual (AV) spatial disparity, ranging from 0° to 20°. First, results in Fig.4, Left Panel show that increasing the strength of cross-modal connections (Wc) consistently reduces the switch cost across all AV disparities, as a result of a stronger cooperation between sensory modalities. While switch cost decreases with increasing AV disparity across all Wc conditions, the rate of reduction is less pronounced at lower Wc values, possibly suggesting a greater sensitivity to disparity in models with stronger cross-modal coupling.**

**Similarly, increasing the effectiveness of lateral intra-area connectivity (Lin) leads to a reduction in switch cost (Fig. 4, Right Panel), with model performance at Lin=5 consistently outperforming that at Lin=3 across disparities. The decline in switch cost with increasing AV disparity is again evident in all conditions, but stronger lateral interactions (higher Lin) sre associated with lower overall switch costs, likely indicating that lateral reinforcement within sensory areas may facilitate faster modality switching.**

~~
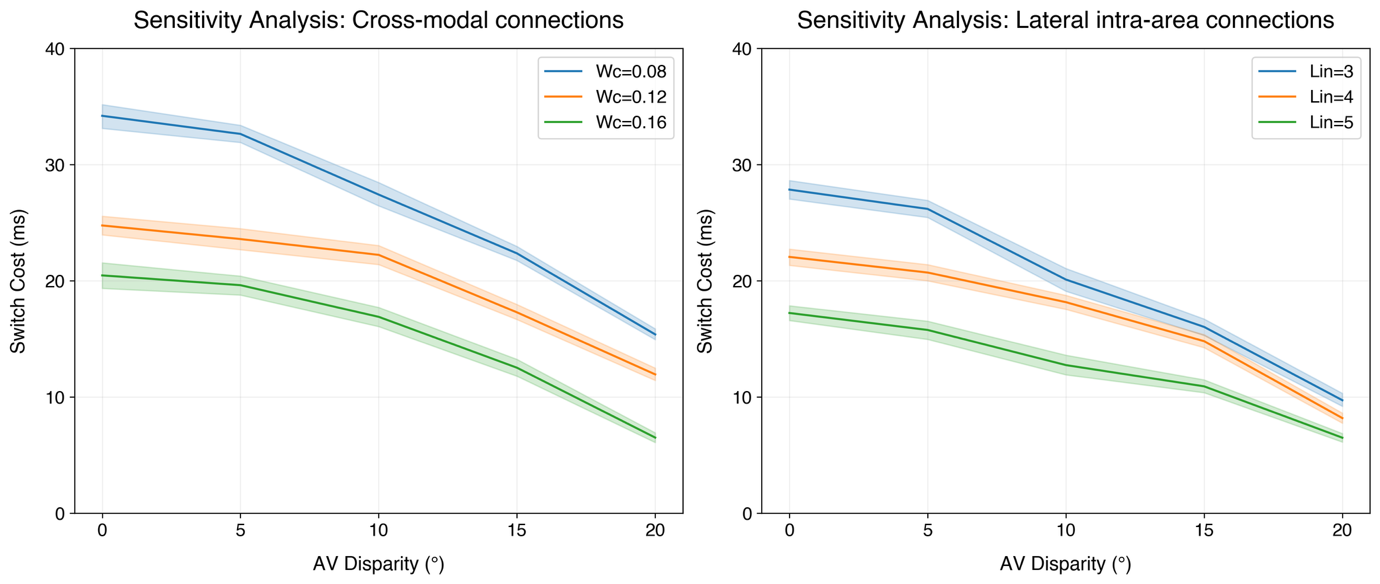
~~**In both cases, shaded areas represent the standard error of the mean, highlighting the consistency of the effect across simulations. These results clearly show that both cross-modal and intra-area connectivity parameters critically influence the model’s capacity for flexible sensory switching, particularly under conditions of low AV spatial disparity.**

**Supplementary Fig. 1: Sensitivity Analyses**. **Left panel**: Effects of varying the strength of **cross-modal connections** (Wc) on switch cost. Increasing Wc from 0.08 to 0.16 leads to a systematic reduction in switch cost across all AV disparities, with the strongest connections (green) yielding the lowest costs. **Right panel**: Effects of varying the strength of the inhibitory component of **lateral intra-area inhibitory connections** (Lin) on switch cost. Higher lateral inhibition (Lin=5) also leads to reduced switch costs, especially at higher disparities. Shaded areas represent standard errors of the mean.

References

Cuppini, C., Magosso, E., Bolognini, N., Vallar, G., & Ursino, M. (2014). A neurocomputational analysis of the sound-induced flash illusion. *NeuroImage*, *92*, 248–266. https://doi.org/10.1016/j.neuroimage.2014.02.001

Jansen, B. H., & Rit, V. G. (1995). Electroencephalogram and visual evoked potential generation in a mathematical model of coupled cortical columns. *Biological Cybernetics*, *73*(4), 357–366. https://doi.org/10.1007/BF00199471
